# A fixation method to preserve cultured cell cytonemes facilitates mechanistic interrogation of morphogen transport

**DOI:** 10.1242/dev.152736

**Published:** 2017-10-01

**Authors:** William J. Bodeen, Suresh Marada, Ashley Truong, Stacey K. Ogden

**Affiliations:** 1 Department of Cell and Molecular Biology, St. Jude Children's Research Hospital, Memphis, TN, 38105, USA; 2 Integrated Program in Biomedical Sciences, University of Tennessee Health Sciences Center, Memphis, TN, 38163, USA

**Keywords:** Cytoneme, Hedgehog, Dispatched, Morphogen, Signal transduction

## Abstract

During development, extracellular cues guiding cell fate determination are provided by morphogens. One mechanism by which morphogens are proposed to traverse extracellular space is by traveling along specialized filopodia called cytonemes. These cellular highways extend between signal-producing and -receiving cells to enable direct morphogen delivery. Although genetic studies support cytoneme involvement in morphogen transport, mechanistic insight into how they are regulated is limited owing to technical challenges associated with performing cell biological analysis of the delicate filopodial structures. Here, we introduce a fixation method whereby cultured cell cytonemes can be preserved for imaging studies, allowing investigation of cytoneme regulation using standard cell biological techniques. Using this method, we examined Hedgehog-containing cytonemes and identified a role for the Hedgehog deployment protein Dispatched in cytoneme stabilization. We demonstrate that Hedgehog and Dispatched colocalize in cytonemes, and that cholesterol-modified Hedgehog acts through Dispatched to increase cytoneme occurrence. Live imaging suggests that this occurs through Dispatched-mediated slowing of cytoneme retraction rates. Dispatched-induced cytoneme modulation was recapitulated in wing imaginal discs of transgenic *Drosophila*, providing evidence that cultured cell cytoneme analysis is predictive of *in vivo* functionality.

## Abstract (precis)

**Summary:** A new fixation method for preserving cultured cell cytonemes, used in combination with live cell imaging, reveals that the Hedgehog deployment protein Dispatched promotes cytoneme occurrence by slowing retraction rates.

## INTRODUCTION

During development, tissue patterning occurs as a consequence of morphogenetic signaling. Morphogens are produced in and deployed from cells localized to discrete domains of developing tissues, and spread from their source to provide instructional cues to signal-receiving cells. Morphogens signal short-range to adjacent cells, and long-range to targets situated at significant distances from the source (Tabata and Takei, 2004). The exact processes facilitating morphogen transport and distribution are not yet fully established. However, several models have been proposed, including free or hindered diffusion, transcytosis, exovesicle shuttling and direct delivery through specialized filopodia called cytonemes (Muller et al., 2013). A putative role for cytonemes in morphogenesis was fortuitously discovered through GFP enhancer trap studies in *Drosophila* wing imaginal discs (Ramírez-Weber and Kornberg, 1999). In this study, threadlike actin-based extensions containing cytoplasmic GFP were observed projecting toward the anterior/posterior (A/P) signaling center of the wing disc, indicating a potential role in cell-cell communication (Kornberg and Roy, 2014; Ramírez-Weber and Kornberg, 1999). Subsequent studies identified Hedgehog (Hh), Wingless (Wg/Wnt), Decapentaplegic (Dpp), epidermal growth factor (EGF) and fibroblast growth factor (FGF) family ligands and receptors localized to the extensions, leading to a model in which the filopodia functioned to deliver signaling molecules directly from sites of production to reception sites (Bischoff et al., 2013; Gradilla and Guerrero, 2013; Hsiung et al., 2005; Huang and Kornberg, 2015; Lidke et al., 2005; Rojas-Ríos et al., 2012; Roy et al., 2011, 2014; Snyder et al., 2015; Stanganello et al., 2015).

A number of recent studies highlight the importance of cytonemes in transport of Hh across *Drosophila* epithelia (Bischoff et al., 2013; Callejo et al., 2011; Chen et al., 2017; Gradilla et al., 2014; Rojas-Ríos et al., 2012). In ligand-producing cells, Hh morphogens are produced as precursor proteins that auto-catalytically cleave to generate a truncated amino-terminal signaling domain. During cleavage, cholesterol is covalently linked to the newly generated carboxyl-terminus of the amino-terminal signaling fragment (Lee et al., 1994). The amino-terminal cysteine is subsequently modified with a long chain fatty acid to produce mature Hh ligand (Chamoun et al., 2001; Long et al., 2015; Pepinsky et al., 1998). The addition of two lipid modifications anchors Hh to producing-cell membranes, necessitating a process by which Hh is deployed from its site of production to establish a morphogen gradient. One protein known to be involved in this process is the 12-pass transmembrane (TM) protein Dispatched (Burke et al., 1999; Ma et al., 2002). Disp loss of function corrupts the Hh gradient to disrupt pathway induction in long-range target cells. This triggers severe developmental defects and early embryonic lethality, underscoring the importance of Disp function for proper Hh ligand dissemination (Caspary et al., 2002; Kawakami et al., 2002; Ma et al., 2002). The exact mechanism by which Disp promotes deployment of lipid-modified Hh to generate its morphogen gradient is not clear. However, consistent with its established role in Hh mobilization, Disp was found to localize to cytonemes oriented towards Hh target cells in *Drosophila* imaginal discs (Gradilla et al., 2014).

To gain insight into Disp function in cytonemes, we sought to use cell biological methods to interrogate cytoneme-based Hh transport in cultured cells. Although protocols for fixation of nanotubes, which share some characteristics with cytonemes, have been reported (Chauveau et al., 2010; Davis and Sowinski, 2008), methods for analyzing fixed cell cytonemes were limited. This has primarily been due to technical challenges associated with cytonemes being very thin (∼200 nm) and easily damaged by standard cell fixation and laser-based imaging techniques (Ramírez-Weber and Kornberg, 1999). To overcome these obstacles, we used a modified electron microscopy fixative, hereafter referred to as MEM-fix, which preserved filopodial structures with cytoneme characteristics for cultured cell imaging studies. This enabled us to utilize standard cell transfection and dsRNA treatment protocols to express or knock down proteins of interest, and assess their effects on functionality of Hh-containing cytonemes.

Here, we report that Hh and Disp colocalize in cytonemes of cultured cells, and that their expression increases cytoneme occurrence. Increased occurrence is dependent upon Disp activity because knockdown of endogenous *disp*, or overexpression of a non-functional Disp mutant, prevents Hh from altering cytoneme behavior. Moreover, elimination of the Hh cholesterol modification, which allows Hh to bypass Disp for deployment (Burke et al., 1999), reduces its cytoneme localization and ablates Hh influence on cytoneme occurrence. Live cell imaging reveals that Disp expression slows cytoneme retraction rates, suggesting that Disp might affect occurrence by stabilizing existing projections. Results obtained from analysis of cultured cells were predictive of *in vivo* functionality. Studies using transgenic *Drosophila* revealed that overexpression of wild-type Disp promoted cytoneme occurrence in wing imaginal disc tissue. Conversely, overexpression of a non-functional Disp mutant failed to enhance cytoneme density, and triggered adult lethality. Combined, these results suggest Disp contributes to Hh transport, at least in part, by influencing cytoneme behavior.

## RESULTS

### Validation of cultured cell cytonemes

**Fig. 1.**
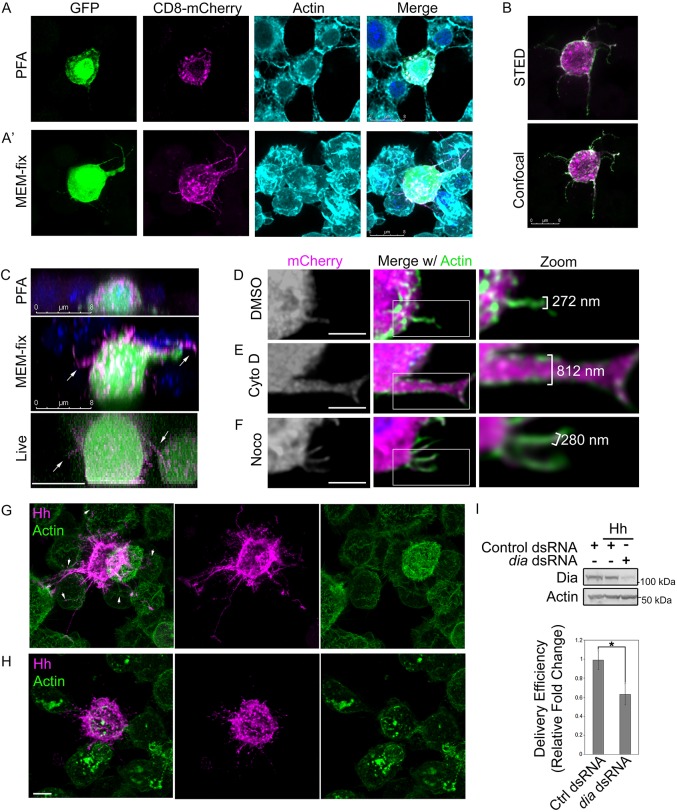
**MEM-fix preserves cultured cell cytonemes.** (A,A′) S2 cells expressing GFP and CD8-mCherry were fixed in 4% PFA (A) or MEM-fix (A′) and stained with phalloidin to label actin. (B) S2 cells expressing Hh (green) and CD8-mCherry (magenta) were MEM-fixed, immunostained for Hh and imaged by STED and confocal microscopy. (C) *z*-stack images of S2 cells expressing GFP and CD8-mCherry were collected from PFA-fixed, MEM-fixed and live cells. DAPI is shown in blue in fixed samples. (D-F) S2 cells expressing cytoplasmic mCherry were plated and treated with DMSO vehicle (D), 10 µm cytochalasin D (E) or 10 µm nocodazole (F) for 1.5 h prior to processing for imaging. Phalloidin (green) marks actin. (G,H) Hh-expressing cells were cultured with non-expressing cells. Hh puncta (magenta) are evident in non-expressing neighboring cells contacted by Hh-positive filopodia (arrowheads). Phalloidin (green) marks actin. (I) More than 60 cells per condition were analyzed over three independent experiments to determine average Hh delivery efficiency to non-expressing cells in contact with a cytoneme. Significance was determined using a two-tailed Student's *t*-test (*P≤0.05). Error bars indicate s.e.m. Lysates from dsRNA-treated cell populations are shown to confirm Dia knockdown. Actin is the loading control. Scale bars: 8 µm (A-C); 5 µm (D-H).

Cytoneme studies were initiated by testing whether MEM-fix (4% paraformaldehyde, 0.5% glutaraldehyde, 0.1 M Sorenson's phosphate buffer, pH 7.4) would preserve thin filopodia for fluorescent microscopy-based analysis of cultured cells better than standard 4% paraformaldehyde (PFA). Glutaraldehyde was added to standard PFA fixative because of its ability to preserve subcellular structures effectively. To overcome the propensity of glutaraldehyde to autofluoresce, sodium borohydride was added to permeabilization buffer to quench autofluorescence prior to antibody incubation. Although glutaraldehyde-based fixatives can hinder protein immunodetection owing to decreased antibody penetration (Bacallao et al., 2006), we reasoned that plasma membrane-localized proteins such as Disp and Hh would be likely to remain accessible for antibody-based detection. However, to overcome this potential limitation during initial testing of MEM-fix, we used fluorescently tagged reporter proteins. *Drosophila* embryo-derived Schneider 2 (S2) cells were transfected with cDNA encoding green fluorescent protein (GFP) and membrane-tethered CD8-mCherry (Roy et al., 2014). S2 cells were selected for analysis based upon their spherical morphology and the ease by which proteins of interest can be knocked down or overexpressed. S2 cells were subjected to PFA or MEM-fix fixation, stained with phalloidin to label actin filaments, and then analyzed by confocal fluorescence microscopy. Actin-containing filopodial structures marked with cytoplasmic GFP and membrane-tethered CD8-mCherry were rarely observed in PFA-fixed samples (Fig. 1A, Fig. S1A). Actin extensions were occasionally present in the PFA-fixed cell population. However, these extensions lacked clear GFP or CD8-mCherry signal, suggesting they were not cytonemes. Conversely, actin-, GFP- and CD8-mCherry-positive extensions reaching between cells were consistently apparent in MEM-fixed samples (Fig. 1A′, Fig. S1A), indicating that cytonemes might be preserved by using the modified fixative. Moreover, the ability to stain actin filaments post-fixation suggested that addition of glutaraldehyde would not eliminate antibody penetration during immunofluorescence-based analysis. To test this, S2 cells were transfected with Hh and CD8-mCherry expression vectors, MEM-fixed, immunostained using anti-Hh, and then imaged by confocal microscopy and stimulated emission depletion (STED) microscopy (Fig. 1B). Hh and CD8-mCherry signals were evident in extended filopodia using both confocal microscopy and super-resolution STED microscopy. As such, MEM-fix does not preclude immunofluorescence analysis.

To determine how the three-dimensional morphology of cells fixed with PFA and MEM-fix compared with what would be observed with live-cell imaging, *z*-stack confocal images of PFA-fixed, MEM-fixed and live S2 cells expressing GFP and CD8-mCherry were acquired (Fig. 1C). For PFA-fixed cells, *z*-stack imaging of the entire cell from base to top revealed an average cell depth of approximately 6.11±0.31 µm. We failed to detect cytoneme-like projections extending from regions of the cell not in contact with the slide in any of the PFA-fixed samples (top). MEM-fixed cells were less compressed than PFA-fixed samples, averaging cell depths of 9.53±0.51 µm from base to top. Cells in MEM-fixed samples frequently showed CD8-mCherry-containing projections originating from the ‘top’ half of the cell body (Fig. 1C, middle panel, arrows). Live image analysis revealed cellular morphologies more similar to MEM-fixed than to PFA-fixed samples. Live cells averaged a depth of approximately 9.9±0.5 µm, and were consistently observed to have CD8-mCherry extensions (Fig. 1C, bottom panel, arrows). As such, MEM-fix preserves S2 cellular dimensions and morphologies more effectively than traditional PFA fixative.

Filopodia are classified as cytonemes if they are actin-based, greater than 2 µm in length, approximately 200 nm thick, and contain morphogens (Ramírez-Weber and Kornberg, 1999). To begin to test whether the observed filopodia met these criteria, we first assessed actin dependence. S2 cells expressing cytosolic mCherry were treated with cytochalasin D to inhibit actin polymerization, nocodazole to block microtubule polymerization or DMSO vehicle control for 1.5 h prior to MEM-fixation and actin staining. Morphology of mCherry- and actin-positive filopodia was examined by confocal microscopy (Fig. 1D-F, Fig. S1B). The architecture of actin-positive filopodia was altered in response to cytochalasin D treatment, as evidenced by increased width of the extensions (Fig. 1E compared with 1D; Fig. S1B). Whereas vehicle-treated cells had filopodia ∼300 nm in width, filopodia of cytochalasin D-treated cells were nearer 800 nm. Nocodazole treatment did not affect filopodial architecture or width (Fig. 1F). These results suggest that whereas actin is required to maintain normal morphology of the cytoplasm-containing extensions, microtubules are not. The filopodia therefore meet the criterion of actin-dependence.

To determine whether GFP- and actin-positive filopodia were of an appropriate length to be considered cytonemes, extensions in confocal images were measured. Based upon analysis of ∼40 labeled filopodia across three experiments, lengths of the extensions were determined to range from approximately 2 µm to 12 µm, with the average filopodial length across the population being 5±0.24 µm. To measure accurately filopodial widths falling below the 200 nm diffraction limit of light microscopy, fluorescent STED microscopy images were examined. Image analysis revealed filopodial widths ranging from approximately 120 nm to 380 nm, with an average width across the filopodial population of 230±75 nm. As such, length and width of the GFP- and actin-containing extensions were consistent with cytoneme size parameters.

We next sought to determine whether Hh-containing filopodia might deliver morphogen to neighboring cells. To test this, Hh-expressing S2 cells were plated on microscopy slides with non-expressing control cells for 1.5 h prior to MEM-fixation. Fixed samples were immunostained for Hh, phalloidin-stained for actin, and then analyzed by fluorescence confocal microscopy. Hh-containing filopodia emanating from Hh-expressing cells were observed to orient towards non-expressing neighbors (Fig. 1G, magenta). Moreover, distinct Hh-positive puncta were detected in cells contacted by filopodia (Fig. 1G, arrowheads), suggesting delivery of Hh through the extensions.

To determine whether the extensions were indeed delivering morphogens, we targeted the Formin homology domain-containing protein Diaphanous (Dia). Dia localizes to tips of *Drosophila* cytonemes where it has been found to affect Dpp morphogen transfer by promoting actin nucleation. Knockdown of *dia* in the Dpp-responsive air sac primordium (ASP) shortens ASP cytonemes and blunts their tips, leading to decreased Dpp signal transduction (Roy et al., 2014). To determine whether Hh delivery from the projections was dependent upon Dia activity, and therefore cytoneme-like, *dia* was knocked down in Hh-expressing cells, and delivery of Hh to non-expressing neighboring cells was tested (Fig. 1H,I). Consistent with reported results (Roy et al., 2014), cytonemes of *dia* knockdown cells appeared shorter, and ends appeared more globular than cytonemes of control dsRNA-treated cells (Fig. 1H versus 1G). Hh delivery efficiency for the two populations was determined by analyzing ∼60-75 cells per condition across three independent experiments, and monitoring for Hh-positive puncta in non-Hh-expressing cells contacted by a cytoneme. *dia* knockdown resulted in a ∼40% reduction in Hh delivery efficiency compared with control (Fig. 1I), supporting the suggestion that the observed filopodia delivered Hh morphogen in a Dia-dependent manner. As such, cultured cell filopodia recapitulate structural and functional characteristics observed with *in vivo* cytonemes.

### Dispatched enriches in cytonemes

**Fig. 2.**
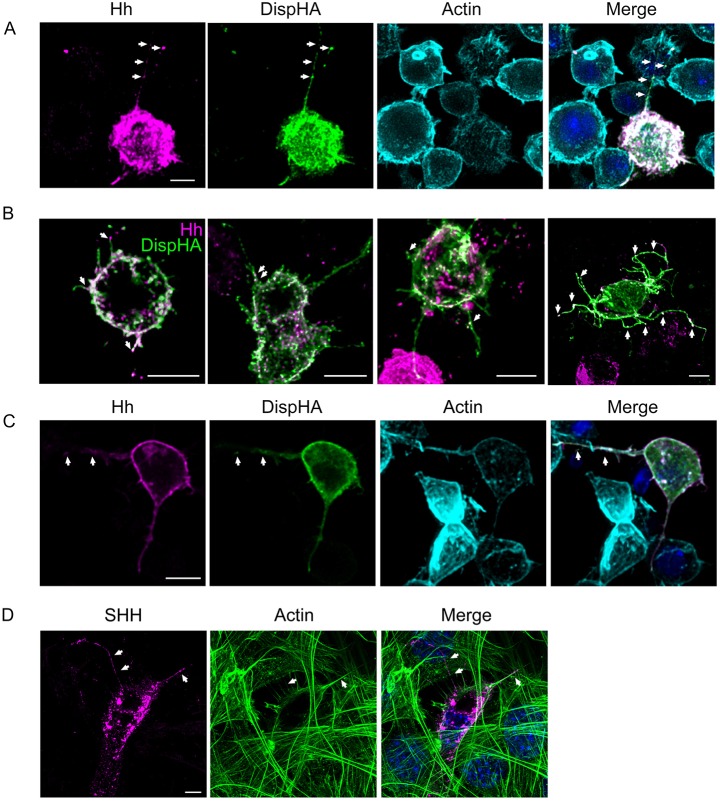
**Disp and Hh localize to cytonemes of cultured cells.** (A-C) DispHA (green) was co-expressed with Hh (magenta) in S2 (A,B) and Cl-8 (C) cells. Cells were MEM-fixed, immunostained and imaged by fluorescence confocal microscopy. Colocalization is indicated by arrows. Phalloidin (cyan) marks actin in A and C. (D) SHH (magenta) was expressed in NIH3T3 cells. Cells were fixed with MEM-fix, stained for actin (green) and imaged by confocal microscopy. DAPI (blue) marks nuclei. Arrows indicate SHH-positive puncta. Scale bars: 5 µm.

As a first step in testing for Disp function in Hh-containing cytonemes, we confirmed that Disp could be detected with Hh in cultured insect cell cytonemes. HA epitope-tagged Disp (DispHA) was co-expressed with Hh, and colocalization between HA and Hh signals was examined in MEM-fixed S2 cells (Fig. 2A). Hh (magenta) localized to puncta on membranes of ligand-expressing cells. DispHA (green) enriched on the plasma membrane and in actin-positive cytonemes, colocalizing with Hh puncta in both plasma and cytoneme membrane domains (Fig. 2A, white arrows). DispHA- and Hh-containing cytonemes varied in number and length across the transfected cell population, but were typically oriented towards neighboring cells (Fig. 2B). Whereas DispHA consistently enriched throughout cytoneme membranes, Hh was largely sequestered to punctate structures (Fig. 2A,B, arrows).

The observed punctate localization of Hh in S2 cell cytonemes was reminiscent of exosomes reported to transport Hh along cytonemes of wing imaginal disc cells and SHH along specialized filopodia in the chick limb bud (Gradilla et al., 2014; Sanders et al., 2013). To test whether similar localization would occur in cultured wing disc-derived cells, Clone 8 (Cl8) cells expressing Hh and DispHA were MEM-fixed and cytonemes were examined. Similar to S2 cells, Cl8 cells produced actin-positive filopodia in which DispHA and Hh colocalized. Although Hh was more enriched along the length of Cl8 cytonemes than it was in S2 cytonemes, discrete Hh puncta overlapping with DispHA were present (Fig. 2C, arrows). To determine whether the same would be true in cultured vertebrate cells, SHH was expressed in murine NIH3T3 cells, and assessed by immunofluorescence confocal microscopy after MEM-fixation. SHH puncta were visible in actin-positive filopodia (Fig. 2D, arrows), confirming that vertebrate cell cytonemes are also likely to be amenable to MEM-fix-based analysis. Moreover, these results suggest conservation of Hh and SHH ‘packaging’ for cytoneme-based transport in cultured cells.

### Hh and Disp increase cytoneme occurrence

**Fig. 3.**
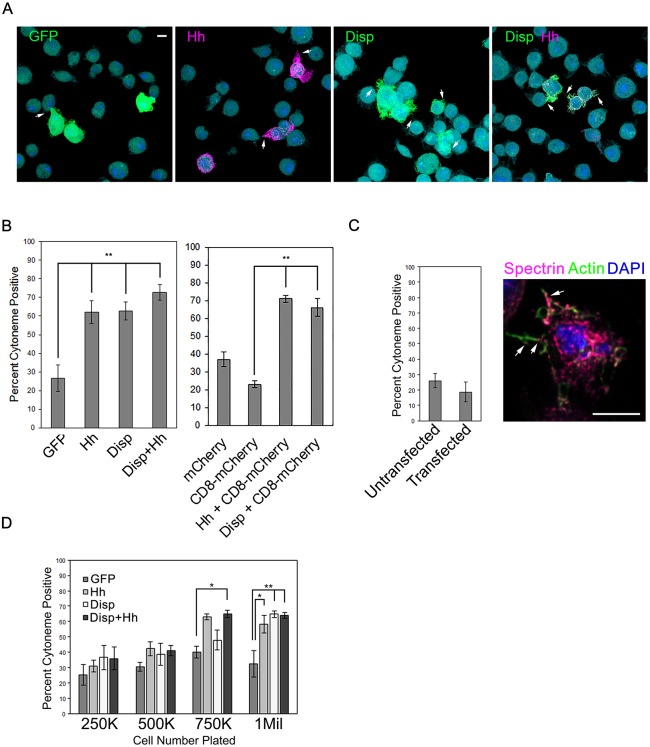
**Disp and Hh increase cytoneme occurrence.** (A,B) S2 cells were transfected with vectors encoding the indicated proteins, and cytoneme occurrence (arrows) in transfected cells was quantified. Statistical significance was determined using a one-way ANOVA (**P≤0.01). Representative fields of cells are shown in A. Approximately 150 transfected cells were counted per condition across three independent experiments. (C) Cytoneme occurrence was quantified in untransfected S2 cells and S2 cells transfected with empty *pAc5.1* vector. Cytonemes were identified by actin (green) and the membrane protein α-Spectrin (magenta, arrows). Percentage of cytoneme occurrence was calculated for approximately ∼150 cells per condition across three separate experiments, and all data were pooled. (D) Cells were plated at increasing densities and cytoneme occurrence was quantified. Approximately 75 cells per condition were counted. The experiment was repeated three times and all data pooled. Significance was determined using a two-way ANOVA (*P≤0.05, **P≤0.01). Error bars indicate s.e.m. Scale bars: 5 µm.

During image analysis of MEM-fixed insect cells, we noticed that cytoneme occurrence was more pronounced in Hh- and DispHA-expressing cells than in control GFP- or CD8-mCherry-expressing populations, indicating that Hh and/or Disp might influence cytoneme dynamics. To test this hypothesis, S2 cells were transfected with plasmids encoding GFP, mCherry, CD8-mCherry, Hh and DispHA, either alone or in combination, and the occurrence of actin-positive cytonemes was quantified (Fig. 3A,B). For this assay, extensions were included in calculations if they contained actin, were ≥2 µm in length and emanated from portions of the cell not in contact with the slide. To ensure that quantification was not skewed by the large number of non-transfected cells in the population, only cells positive for signal of the transfected protein(s) were included in calculations. Expression of Hh, DispHA, or the two together resulted in a significant increase in the percentage of transfected cells extending cytonemes towards neighboring cells compared with GFP- or CD8-mCherry-expressing controls. Whereas ∼20-25% of GFP- and CD8-mCherry-positive cells analyzed had cytonemes, ∼60% of Hh-expressing cells had them. The percentage occurrence observed in DispHA-expressing cells and cells expressing DispHA along with Hh was similarly increased. Importantly, DispHA-induced cytoneme increases over control were similar whether comparing with GFP- or CD8-mCherry-expressing control cells, indicating that the ability to visualize and score cytonemes was not dependent upon or skewed by expression of a membrane protein.

To confirm that occurrence rates were not influenced by lipid-based transfection of cDNA expression vectors, cytoneme occurrence was determined in *pAc5.1* empty vector-transfected S2 cells, and compared with occurrence rates of untransfected cells. Fixed cells were immunostained for endogenous α-Spectrin to mark cell membranes (Dubreuil et al., 1997), and stained with phalloidin to mark actin (Fig. 3C). Quantification of actin- and α-Spectrin-positive cytonemes showed average occurrence rates of ∼20-25% in both transfected and untransfected cell populations, ruling out changes in cytoneme behavior resulted from lipid-based plasmid transfection.

In *Drosophila*, cytoneme outgrowth is promoted by the fly FGF Branchless (Roy et al., 2014). The ability of Hh to promote cell proliferation could lead to increased cell density in cultures, potentially resulting in higher amounts of FGF in culture media capable of promoting cytoneme occurrence. To test directly whether cell density would affect cytoneme occurrence in control and Hh/Disp-expressing cells, S2 cells were transfected with vectors encoding GFP, Hh and/or DispHA, and then 2 days later plated at increasing densities on chamber slides. Densities ranged from 250,000 cells/well of a 4-well slide (very sparse) to 1×10^6^ cells per well, which corresponds to the density used in functional assays. Approximately 75 cells per density were counted across three independent experiments, and the average percentage of cytoneme-positive cells was quantified as described above. Cytoneme occurrence in GFP-expressing control cell populations ranged from ∼25-40% across the plating densities tested (Fig. 3D, dark gray). Although increased cell density trended towards increased cytoneme occurrence, the increases observed across the GFP populations failed to reach statistical significance at any density tested. Expression of DispHA (white bars), Hh (light gray) or the two proteins together (black) did not significantly increase cytoneme occurrence at the lower plating densities (250,000-500,000 cells/well). However, statistically significant increases over the GFP-expressing control cell occurrence rates emerged at higher plating densities (750,000-1×10^6^ cells/well). These results suggest that (1) increased cell density is not sufficient to significantly increase cytoneme occurrence in cultured cells, and (2) cell density must reach a critical threshold to allow for Hh- and Disp-induced cytoneme effects to occur. Thus, increased cell density is necessary, but not sufficient, for Hh- and Disp-mediated alteration of cytoneme occurrence.

**Fig. 4.**
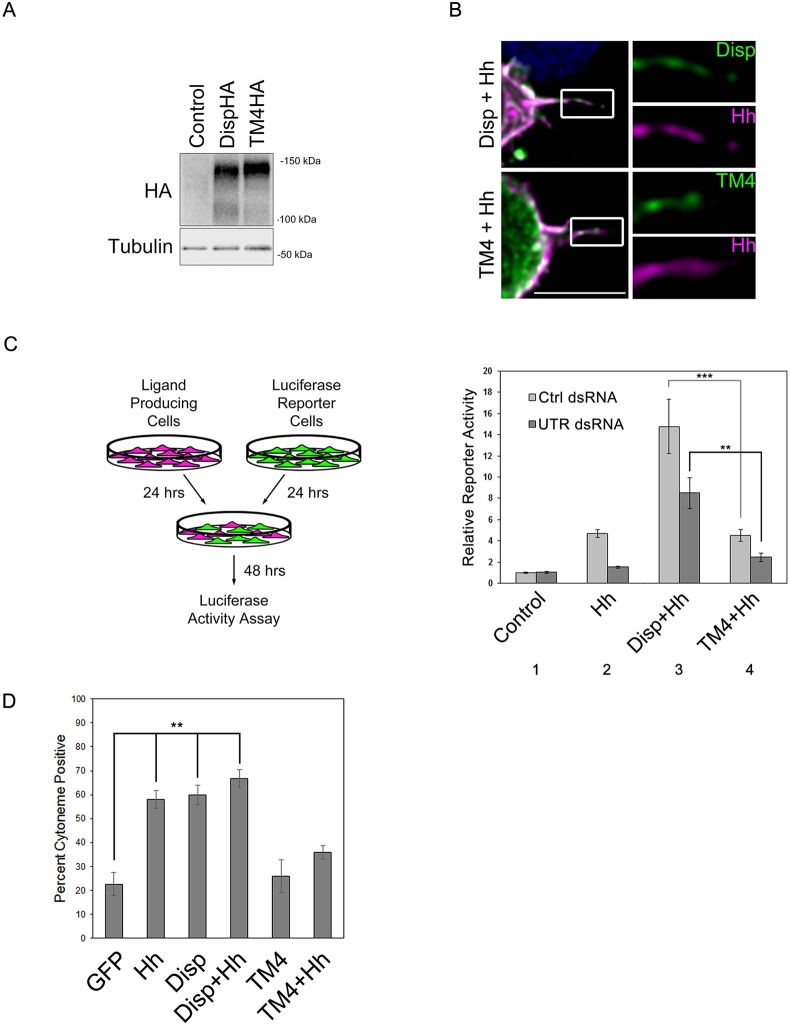
**Cytoneme modulation correlates with Hh deployment.** (A) Lysates were prepared from Cl8 cells expressing wild-type or TM4 DispHA proteins, and analyzed by western blot. Tubulin is the loading control. (B) S2 cells expressing wild-type or TM4 DispHA proteins (green) along with Hh (magenta) were fixed in MEM-fix, immunostained and imaged by confocal fluorescence microscopy. Scale bar: 5 µm. (C) Cl8 reporter cells transfected with *ptcΔ136-luciferase* reporter and *actin-Renilla* normalization control were co-cultured with Hh-producing cells in which *disp* expression was modulated. Ligand-producing cells were treated with control or *disp3′UTR* dsRNA, and transfected with empty vector control or plasmids encoding wild-type or the TM4 Disp proteins. The experiment was repeated two times in duplicate and all data pooled. Error bars indicate s.e.m. Statistical significance was determined using a one-way ANOVA (**P≤0.01, ***P≤0.001). (D) To determine cytoneme occurrence rates, approximately ∼150 cells per condition were examined over three separate experiments and all data were averaged as in Fig. 3. Error bars indicate s.e.m, (**P≤0.01).

Both Disp and Hh localize to the plasma membrane, raising the possibility that altered cytoneme occurrence could be the result of overexpression of a functional membrane protein. An established Disp transporter mutant, which contains D-to-A alterations of conserved residues in TM domains 4 and 10 (D516A, D517A, D1030A), has been demonstrated to disrupt Hh transport (Callejo et al., 2011; Ma et al., 2002). To confirm that cytoneme occurrence in our system would correlate with the ability to transport Hh, and not with overexpression of a membrane protein, we attempted to express this mutant in S2 cells and calculate cytoneme occurrence rates. Unfortunately, the TM4/TM10 transporter mutant was unstable when expressed in S2 cells (data not shown). We therefore disrupted the TM4/TM10 transporter motif by targeting TM4 residues D516-D517. Western blot of lysates from wild-type and DispTM4HA-expressing cells showed similar Disp protein levels (Fig. 4A). Immunofluorescence analysis revealed that DispTM4HA enriched on the cell surface and could localize to cytonemes, indicating that its cellular trafficking was intact (Fig. 4B).

To confirm that DispTM4 was non-functional for Hh deployment, co-culture Hh reporter assays were performed. S2 cells do not express the Hh transcriptional effector Ci, so for these experiments Cl8 cells were used. Hh-responsive *ptcΔ136-luciferase*/*pAc-Renilla* reporter cells (green) were co-cultured with ligand-producing cells (magenta) in which endogenous *disp* was knocked down in the presence or absence of wild-type or TM4 DispHA cDNA (Fig. 4C). Reporter cells co-cultured with Hh-producing cells expressing endogenous *disp* and treated with control dsRNA showed a ∼5-fold increase in reporter activity over baseline, consistent with Hh-producing cells effectively deploying Hh to target cells (light gray bars, columns 1-2). This response was ablated by treating Hh-producing cells with dsRNA directed at the 3′UTR of the endogenous *disp* gene (column 2, dark gray bar). Introduction of wild-type DispHA cDNA lacking UTR sequence into the ligand-producing cells enhanced the Hh response in the control dsRNA-treated population and rescued loss of signal in the *disp* knockdown population (column 3). Conversely, the DispTM4HA mutant was unable to rescue *disp* knockdown in *disp 3′UTR* dsRNA-treated cells. Moreover, overexpression of this mutant in the control dsRNA background did not enhance activity as was seen with wild-type DispHA (column 4 compared with 3), confirming that targeting TM4 is sufficient to disrupt Disp-mediated Hh deployment.

We next tested the effect of non-functional DispTM4HA on cytoneme occurrence. Unlike what was observed for wild-type DispHA, DispTM4HA did not enhance cytoneme occurrence, and instead appeared to function in a dominant-negative manner, ablating the Hh-induced cytoneme increase (Fig. 4D). Disp has been demonstrated to function as a trimer (Etheridge et al., 2010), suggesting that DispTM4HA could function in a dominant-negative manner to block function of endogenous Disp protein. Nevertheless, these results confirm that overexpression of a membrane-localized protein is not sufficient to alter cytoneme occurrence, and that the ability of Disp to enhance cytonemes correlates with its ability to deploy ligand.

### Cytoneme modulation requires Hh cholesterol modification

**Fig. 5.**
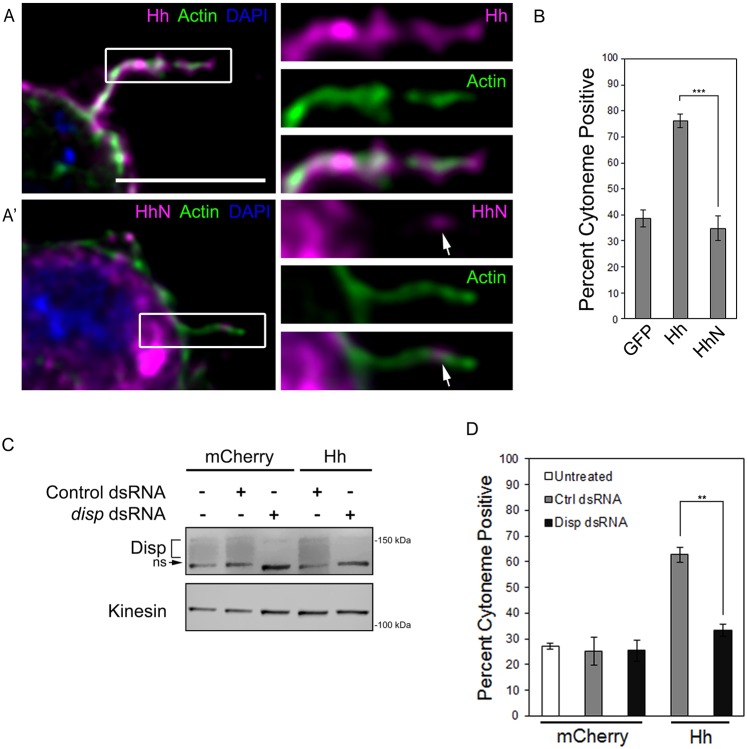
**Hh requires Disp to modulate cytonemes.** (A,A′) S2 cells expressing cholesterol-modified Hh (A, magenta) or cholesterol-free HhN (A′, magenta) were fixed using MEM-fix, immunostained and imaged by confocal fluorescent microscopy. Phalloidin (green) marks actin. DAPI (blue) marks nucleus. Arrows indicate HhN present in the cytoneme. Scale bar: 5 µm. (B) Cytonemes were quantified in S2 cells expressing the indicated proteins. Cytoneme occurrence was quantified in ∼75 cells per condition over three independent experiments and all data pooled. Error bars indicate s.e.m. Significance was determined using a one-way ANOVA test (***P≤0.001). (C) Endogenous *disp* was knocked down in control, cytoplasmic mCherry- or Hh-expressing S2 cells. Kinesin serves as loading control. Disp is detected as a broad band (bracket). A non-specific band (ns) recognized by the antibody is indicated. (D) S2 cells were transfected with Hh or cytoplasmic mCherry expression vectors in the presence of control or *disp3′UTR* dsRNA. Cytoneme occurrence was determined as in B. Error bars indicate s.e.m. Significance was determined using a one-way ANOVA (**P≤0.01).

Expression of cDNA encoding full-length Hh in cultured insect cells generates a Hh protein that is fully processed to its dually lipid-modified form. Processing can be bypassed by expressing cDNA encoding the amino-terminal signaling fragment HhN (Burke et al., 1999; Porter et al., 1996). Because the carboxyl-terminal cholesterol is added during processing, HhN lacks a cholesterol modification, but remains amenable to amino-terminal fatty acid acylation (Chen et al., 2004; Gallet et al., 2006). Although this amino-terminal fragment is competent to induce signaling in ligand-receiving cells *in vitro*, it does not establish a proper morphogen gradient *in vivo* (Gallet et al., 2006; Li et al., 2006). This may be because, unlike Hh, HhN does not require Disp for mobilization from membranes of ligand-producing cells (Burke et al., 1999). The vertebrate HhN ortholog SHH-N has been observed in cytoneme-like filopodia (Sanders et al., 2013). To determine whether *Drosophila* HhN lacking its cholesterol modification would localize to cytonemes, we expressed Hh or HhN in S2 cells and analyzed ligand localization and cytoneme occurrence. Notably, HhN failed to enrich in cytonemes like the lipid-modified ligand, but weak HhN cytoneme signal was occasionally visible in the filopodia (Fig. 5A,A′, arrow). Consistent with failure to localize effectively to cytonemes, HhN did not alter cytoneme behavior. HhN-expressing cells showed occurrence rates similar to GFP-expressing control cells, a ∼40% reduction from occurrence with the cholesterol-modified ligand (Fig. 5B). As such, the Hh cholesterol modification is essential for its ability to enrich in cytonemes and modulate their occurrence.

Based upon the results described above, we hypothesized that the cytoneme effects conferred by expression of cholesterol-modified Hh (Fig. 3A,B, Fig. 4D, Fig. 5B) resulted from its interaction with endogenous Disp. To test this, endogenous Disp was knocked down using 3′UTR-specific dsRNA (Fig. 5C), and the ability of Hh to increase cytonemes in the *disp* knockdown population was tested (Fig. 5D). Quantification of cytoneme occurrence in untreated and control dsRNA-treated mCherry-expressing cells revealed similar cytoneme incidence, indicating that dsRNA treatment did not alter cytoneme occurrence. As expected, expression of Hh in control dsRNA-treated cells increased cytoneme occurrence to ∼60% (gray). Hh failed to affect cytonemes in *disp3′UTR* dsRNA-treated cells (black), indicating that increases in cytoneme occurrence triggered by Hh expression were dependent upon endogenous Disp. Given that S2 cells do not express endogenous *hh* (modENCODE; data not shown), and Disp can modulate cytonemes when expressed without Hh, it is unlikely overexpressed Disp requires endogenous Hh ligand to increase cytoneme occurrence.

### Disp expression correlates with slower cytoneme retraction

Cytoneme outgrowth and retraction rates are highly dynamic, and are modulated by regulatory proteins to affect cytoneme duration (Bischoff et al., 2013). We hypothesized that Disp might confer its effects by altering dynamics to either accelerate cytoneme outgrowth or to prevent retraction. To test this hypothesis, cytoneme durations were calculated from live imaging of S2 cells expressing GFP plus CD8-mCherry control (*n*=23) or Disp-mCherry (*n*=12) (Movies 1 and 2, Table 1). Cytoneme growth and retraction were punctuated by short lags during which no outward or inward movement could be detected. To determine speeds of active movement during growth and retraction accurately, lags were not considered when calculating growth or retraction rates, but were included in total cytoneme duration times. Cytonemes of GFP/CD8-mCherry-expressing control cells showed an average duration of approximately 77.5±7.9 s. In this population, cytoneme growth rate averaged 6.0±0.6 µm/min, and retraction averaged 7.5±1.0 µm/min. Expression of Disp-mCherry increased cytoneme durations nearly 3-fold to an average of ∼234.5±33.1 s. This was likely due to slowing of the retraction rate, which was reduced by approximately 45% to 3.98±0.67 µm/min in Disp-mCherry-expressing cells. The outgrowth rate remained largely unchanged in Disp-mCherry cells, averaging 6.5±0.87 µm/min. Thus, Disp may promote cytoneme occurrence by stabilizing outgrown filopodia. Tracking of Disp-mCherry puncta during cytoneme live imaging revealed Disp trafficking towards cytoneme tips (Movie 3, Fig. S2), raising the possibility that its expression might influence ligand release and/or cytoneme duration through modulation of tip activity.

**Table 1.**
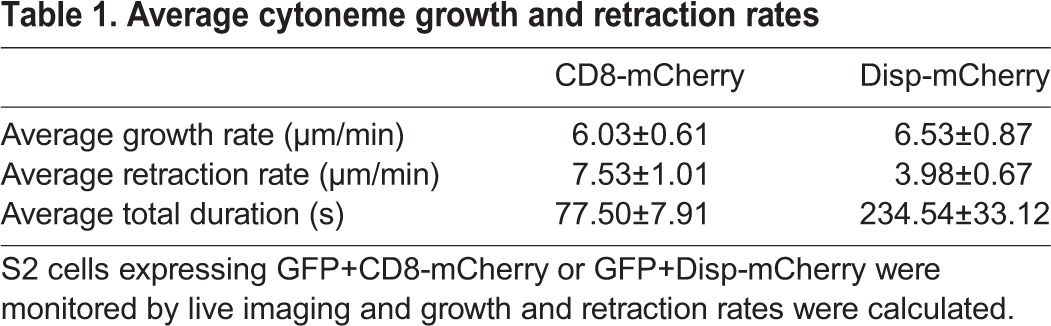
**Average cytoneme growth and retraction rates**

### Studies using cultured cell cytonemes are predictive of *in vivo* behavior

**Fig. 6.**
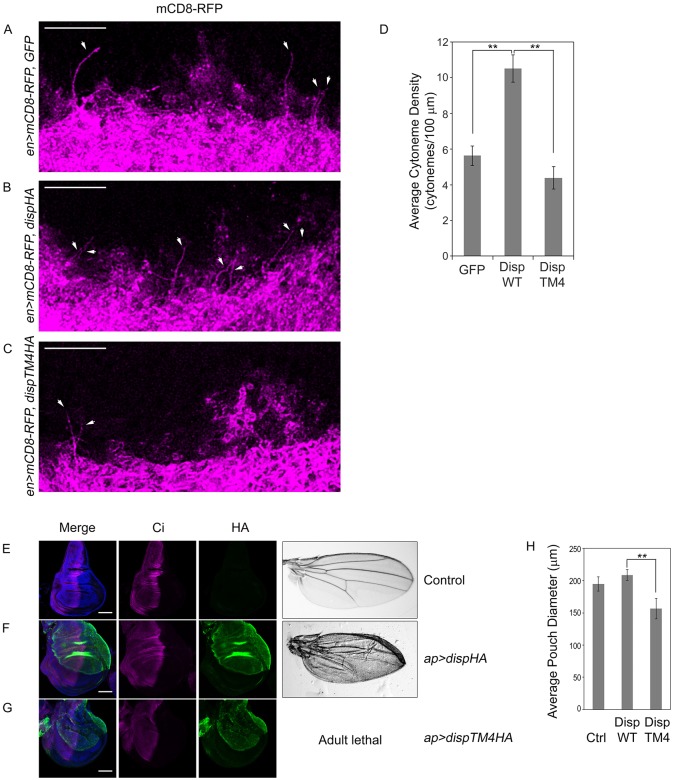
**Disp influences cytonemes *in vivo*.** (A-C) GFP or the indicated DispHA proteins were expressed with CD8-RFP (magenta) in the posterior compartment of wing imaginal discs using *en-GAL4*. The basolateral region of the anterior/posterior border directly adjacent to the dorsal/ventral border was analyzed. Cytonemes reaching into the anterior compartment are indicated (arrows). Six to ten discs were examined per condition. Representative discs are shown. Scale bars: 10 µm. Discs are oriented such that anterior is up. (D) Cytonemes crossing the compartment boundary in a straight posterior to anterior trajectory were counted across multiple discs (GFP *n*=6, DispWT *n*=10, DispTM4 *n*=10). Density was calculated as the average number of cytonemes reaching at least one cell diameter into the anterior compartment, and is presented as average cytoneme number per 100 µm. Error bars indicate s.e.m. Significance was determined using a one-way ANOVA (**P≤0.01). (E-G) DispHA proteins (green) were expressed in the dorsal compartment of wing imaginal discs using *ap-GAL4*. Discs are oriented such that the dorsal compartment is up and anterior is left. Ci is shown in magenta, DAPI in blue. Wings shown on the right are representative of adult phenotypes observed. Scale bars: 50 µm. Average wing pouch size is indicated in H. Error bars indicate s.e.m. Student's *t*-test was used to determine the statistical significance (**P≤0.01).

Studies of cultured cell cytonemes revealed a positive correlation between Disp expression and cytoneme occurrence. To determine whether these results were predictive for what would occur *in vivo*, we tested the effect of DispHA overexpression on wing imaginal disc cytonemes*.* mCD8-RFP was expressed in wing imaginal discs in combination with GFP control, DispHA or DispTM4HA using the UAS/GAL4 system. Wing discs from third instar larvae were dissected and MEM-fixed, and cytonemes were examined by confocal microscopy (Fig. 6A-C). Transgenes were expressed using the posterior-specific *engrailed* (*en*)*-Gal4* driver to allow for clear detection of CD8-RFP-positive cytonemes originating from posterior cells and reaching into anterior tissue. Discs were analyzed by confocal imaging of the basal surface directly adjacent to the intersection of the A/P and dorsoventral boundaries. In a previous study, it was reported that cytonemes could be detected in ∼60% of CD4-Tomato-expressing wing imaginal discs fixed with 4% PFA (Bischoff et al., 2013). In our experiments, cytonemes were evident in all MEM-fixed discs examined, regardless of genotype, suggesting that the modified fixation procedure might also improve ability to detect cytonemes in intact tissue. Numerous long cytonemes marked with mCD8-RFP were evident in MEM-fixed discs crossing the A/P boundary in both control and DispHA-expressing discs, with an apparent density increase occurring in response to DispHA expression (Fig. 6A,B). Conversely, wing discs expressing DispTM4HA showed a decreased density of cytonemes reaching into the anterior compartment (Fig. 6C). To examine these effects quantitatively, cytoneme density was calculated based upon the number of long cytonemes reaching from the posterior compartment in a straight trajectory across the A/P boundary. Although mCD8-RFP-positive extensions were present throughout the discs, and observed to orient in all directions, we focused on those crossing the A/P boundary to simplify quantitative analysis. Quantification revealed that, as was observed in S2 cell experiments, DispHA overexpression triggered a statistically significant increase in cytoneme density (Fig. 6D). Cytoneme density was modestly reduced, but not significantly altered from control in DispTM4HA-expressing discs. Combined, these results support the suggestion that the cytoneme effects observed following modulation of Disp expression in S2 cells were indeed predictive for what would occur *in vivo* (Fig. 3B, Fig. 4D, Fig. 6D).

To determine whether TM4-induced cytoneme depletion correlated with altered *in vivo* patterning, wild-type and TM4 DispHA proteins were expressed in the dorsal compartment of the wing imaginal disc using *apterous* (*ap*)*-GAL4*, and larval discs and adult wings were examined. *ap-GAL4* was selected because dorsal compartment-specific transgene expression allows for direct comparison of affected dorsal tissue and control ventral tissue in the same imaginal disc (Blair et al., 1994; Marada et al., 2016). Wing imaginal discs were dissected from wandering third instar larva, fixed using 4% PFA, immunostained for HA (green) and the full-length form of the pathway transcriptional effector Ci (magenta), and then imaged by confocal microscopy (Fig. 6E-G). Although pronounced effects on Ci stabilization were not evident following overexpression of wild-type DispHA, significant blistering did occur in adult wings (Fig. 6F compared with 6E; Fig. S3A). This phenotype is similar to what has been observed following high-level dorsal overexpression of activating mutants of the Hh signal transducing protein Smoothened, and probably results from overgrowth of dorsal wing blade tissue (Marada et al., 2013). Overexpression of DispTM4HA in the dorsal wing disc triggered larval lethality, with only a few larva surviving to late third instar. Although pronounced effects on Ci stabilization were not evident in escaper wing discs, the discs were consistently reduced in size, and showed malformations of varying severity (Fig. 6G,H, Fig. S3B). Effects on adult wings could not be determined due to adult lethality. As such, cytoneme density alteration in the wing disc correlates with developmental abnormality.

## DISCUSSION

In the current study, we performed cytoneme imaging studies on cultured *Drosophila* cells using a modified electron microscopy buffer we call MEM-fix. Fixation of cells using MEM-fix preserved the delicate cellular structures, allowing for analysis of cytoneme occurrence and regulation following manipulation of proteins of interest through standard cell biological methodologies. We focused on cytoneme-mediated delivery of the Hh morphogen, and identified a novel cytoneme-stabilizing effect resulting from Disp expression. Analysis of transgenic *Drosophila* confirmed that the *in vitro* results obtained using cultured cells were predictive of *in vivo* behavior. As such, analysis of cultured cells may represent a system in which mechanistic interrogation of cytoneme function can be expedited to provide insight into *in vivo* cytoneme regulation.

An instructional role for Hh in regulation of cytonemes was proposed in the first report linking cytonemes with morphogen signaling. In this study, Hh was found to be necessary, but not sufficient, for directional cytoneme growth in wing imaginal disc tissue (Ramírez-Weber and Kornberg, 1999). Subsequent *in vivo* studies revealed that transient overexpression of Hh was not sufficient to modify cytoneme number or length in eye or wing imaginal discs, or in the tracheal ASP, further suggesting that Hh must work in conjunction with other factors to influence cytoneme dynamics (Roy et al., 2011). The Hh co-receptor Interference Hedgehog (iHog) and the Hh transporter protein Disp localize to cytonemes (Bischoff et al., 2013; Callejo et al., 2011), raising the possibility that Hh-binding transmembrane proteins are the conduits linking ligand to cytoneme behavior. Consistent with this notion, iHog appears to stabilize cytonemes when overexpressed in the wing disc (Bischoff et al., 2013). The current study provides additional data supporting the hypothesis, demonstrating that cholesterol-modified Hh increases cytoneme occurrence in a Disp-dependent manner. The ability of Hh to increase cytoneme occurrence in cultured S2 cells was ablated by knockdown of endogenous *disp* and by overexpression of a non-functional Disp mutant. Overexpression of wild-type Disp in S2 cells, which do not express endogenous *hh* (modENCODE; data not shown), was sufficient to increase cytoneme occurrence. Disp expression also promoted cytonemes *in vivo*; its overexpression in wing imaginal discs led to increased density of long cytonemes at the A/P boundary and to wing tissue over-growth in adult flies.

Elucidation of the precise mechanism by which Disp expression alters cytoneme behavior to increase occurrence rates requires further study. However, live-imaging analysis of Disp-expressing S2 cells suggests that stabilization occurs, at least in part, through maintenance of outgrown cytonemes. Based upon the observed movement of Disp-mCherry towards cytoneme tips, we speculate this might occur through Disp directly or indirectly altering actin polymerization at the tip to delay filopodial retraction. Given that Hh is likely released from cytoneme tips, the ability to delay tip retraction would be an advantageous functionality for Disp. Potential mechanisms by which Disp could possibly delay tip retraction include modulation of Formin activity, alteration of tip adhesion and/or regulation of cytoneme-localized motor proteins transporting actin regulators (Bornschlogl et al., 2013; Mellor, 2010).

It is unlikely that Disp functions in isolation to modulate cytoneme behavior. Cultured cell experiments revealed that cell density had to reach a crucial threshold to allow for Disp and Hh to promote cytoneme occurrence. This may be indicative of neighboring cells producing distance-limited cytoneme regulatory signals to provide directional, attractive or repulsive cues to Hh- and/or Disp-containing extensions. Consistent with this notion, FGF is known to promote directional cytoneme outgrowth *in vivo* (Ramírez-Weber and Kornberg, 1999; Roy et al., 2014). Thus, multiple signals sent and received between neighboring cells probably converge to direct cytoneme function during tissue development. Future mechanistic studies using cultured cell cytonemes will be directed at defining this biology, and identifying mechanisms by which Disp might help to integrate such signals and promote function of Hh-containing cytonemes.

In conclusion, by using MEM-fix, a modified electron microscopy fixative, we were able to interrogate cultured cell cytonemes using fluorescent microscopy, and begin to provide insight into how Hh and Disp influence cytoneme behavior. The experimental flexibility provided by cultured cells, coupled with the ability of MEM-fix to preserve their cytonemes, will likely advance functional analysis of cytonemes for additional morphogen classes. Cell culture systems have the potential to accelerate studies addressing provocative questions about cytoneme biology, including how morphogens are gated into cytonemes, how cytoneme-localized proteins are appropriately trafficked within the structures, and how cytoneme directionality and length are controlled to assure morphogens reach their desired targets. Answering such questions will be essential to advance our understanding of how morphogen gradients are generated and reinforced during tissue development.

## MATERIALS AND METHODS

### Cell lines

NIH3T3 (ATCC CRL-1658) and S2 (ATCC CRL-1963) cell lines were obtained from ATCC and Cl8 cells from *Drosophila* Genomics Resource Center (DGRC) (CME W1 Cl.8+). Cell lines were routinely authenticated by functional assay and screened for contamination by PCR. S2 cells were cultured in Schneider's *Drosophila* media (Sigma) plus 10% heat-inactivated fetal bovine serum (FBS) and 1× penicillin/streptomycin (Sigma). Cl8 and NIH3T3 cells were cultured using standard methods.

### Cell fixation and imaging analysis

For fixed cell microscopy, 4×10^6^ S2 or Cl8 cells were plated into 60 mm dishes the day before transfecting with 5 µg of *pAc-GFP*, *pAc-dispHAWT*, *pAc-dispHATM4*, *pAc*-*hh* or *pAc*-*hhN* using Lipofectamine 2000 (Thermo Fisher Scientific). DNA content was normalized using empty *pAc5.1* vector. Forty-eight hours post-transfection, cells were re-suspended by pipetting, and ∼1 million cells/well were plated into chamber slides (LabTek). After 1.5 h incubation, adherent cells were washed three times with 1× PBS, then fixed at 4°C for 7 min using fresh MEM-fix [0.1 M Sorensen's phosphate buffer (pH 7.4), 4% formaldehyde (Polysciences) and 0.5% glutaraldehyde (Electron Microscopy Sciences)]. Fixed cells were washed three times using 1× PBS for 5 min, and then permeabilized for 1 h at room temperature in 0.1% Triton X-100, 5% goat serum (Jackson ImmunoResearch), 1 mg/ml of sodium borohydride. Cells were then incubated in 0.1% Tween-20 and 5% goat serum in 1× PBS containing anti-HA.11 (1:250; Covance), anti-Hh (1:100; SCBT), and phalloidin (Actin Green or Actin Red, Invitrogen) overnight at 4°C in the dark. The next morning, cells were washed three times for 5 min in 1× PBS. Secondary antibodies were incubated in staining buffer at a 1:1000 dilution for 1 h at room temperature. Cells were washed five times for 10 min each in 1× PBS, then mounted using ProLong Gold with DAPI or ProLong Diamond (Invitrogen).

NIH-3T3 cells were seeded at a density of 1×10^6^ cells/60 mm dish in DMEM plus 10% FBS and 1× penicillin/streptomycin (Sigma). Empty *pCDNA3* (2 μg) or *pCDNA3-Shh* (2 μg) was transfected into using Fugene 6 (Promega).

Fixed cells were imaged on Zeiss LSM 780, 3i Marianas Spinning Disc or a Leica TCS SP8 3D STED confocal microscopes. Deconvolution was performed on images using the Huygens Professional software package using Classic Maximum Likelihood Estimation (CMLE) deconvolution calculations with five iterations using slice-by-slice mode. Images were processed using Adobe Photoshop CS6.

For wing disc cytoneme analysis, discs were collected from third instar larva and fixed using MEM-fix for 15 min at room temperature before washing, staining and imaging as above. Primary antibody incubation was performed overnight at 4°C with gentle rocking. Secondary antibodies were incubated for 1 h at room temperature with gentle rocking. Discs were mounted directly on coverslips in ProLong Gold or ProLong Diamond mounting media (Invitrogen). Cytoneme density was calculated by counting the number of long cytonemes crossing the A/P compartment boundary in a straight posterior-to-anterior trajectory in dorsal and ventral regions of the wing pouch. Cytoneme numbers were divided by the length of the dorsal and ventral domains of the wing pouch along the A/P boundary, and plotted as average cytoneme number per 100 µm. For analysis of Ci and Disp expression, wing imaginal discs were fixed in 4% paraformaldehyde for 10 min and analyzed as described (Carroll et al., 2012). Antibodies for immunostaining were used at a concentration of 1:40 for anti-Ci (DSHB) and 1:200 for anti-HA (Roche). Representative discs are shown. To assess wing disc size, diameters of six to eight wing pouches were measured using Zeiss ZEN software.

### Cytoneme quantification

For occurrence rate determination, cells having cytonemes meeting the following criteria were included: filopodia were (1) actin-positive, (2) ≥2 µm in length, (3) ≤500 nm thick, (4) originating from regions of cell membrane not in contact with the slide, and (5) not targeted back to the cell of origin. All cytoneme-positive cells in the transfected population were included in calculations. Occurrence rates were confirmed by a second lab member analyzing results in a blinded fashion. Statistical significance was determined as appropriate as indicated in the figure legends.

### Reporter assays

Hh-producing cells were generated by transfecting 4×10^6^ Cl8 cells in a 60 mm plate with 5 μg control or *disp3′UTR* dsRNA plus indicated expression vectors. To generate reporter cells, a 10 cm dish containing 1×10^7^ Cl8 cells was transfected with 600 ng *ptcΔ136-luciferase* and 60 ng *pAc-Renilla*. Hh-producing and reporter cells were collected ∼24 h post-transfection, re-suspended in fresh media and re-plated into 12-well dishes at a 1:3 ratio of reporter to producing cells. After ∼48 h co-culture reporter assays were performed using Dual Luciferase Assay (Promega) (Carroll et al., 2012).

### Protein knockdown

*disp* 3′ UTR dsRNA was amplified from *pFLC-I-disp* using T7 primers forward (5′-GAATTAATACGACTCACTATAGGGAGATATGCTGGCGGTG) and reverse (5′-GAATTAATACGACTCACTATAGGGAGAGGGATATAACACTATGTCTG) using Phusion High-Fidelity DNA Polymerase (NEB). *dia* dsRNA was generated by amplifying sequence from *pBSK-dia* (DGRC) using T7 primers forward (5′-GAATTAATACGACTCACTATAGGGAGAGCAGACATTGCTGCACTACC) and reverse (5′-GAATTAATACGACTCACTATAGGGAGACCAACAGACTGTCCATCACG). Control dsRNA was generated from *Xenopus* Elongation Factor provided with the T7 Megascript Kit (Thermo Fisher Scientific).

Approximately 4×10^6^ S2 cells were plated onto 60 mm dishes, and the following morning were either bathed with 15 µg of control, *disp3′UTR* or *dia* dsRNA in 4.5 ml serum-free culture media with media/dsRNA replenishment after 5 days or transfected with *pWIZ-empty* or *pWIZ-dia-RNAi* (Roy et al., 2014) every five days for a total of 10 days before transfecting with 5 µg of *pAc5.1-hh* or empty vector control. After 10 days, cells were lysed in 1% NP-40, 150 mM NaCl, 50 mM Tris base, 50 mM NaF, 1× Complete Protease Inhibitor Cocktail (PIC, EDTA-free, Roche), and 0.5 mM DTT, and centrifuged for 5 min at 2000 ***g***. Supernatants were analyzed by western blot for Dia detection. For Disp, cells were lysed by dounce homogenization in 20 mM Hepes, 10 mM KCl, 150 mM NaCl, 1× PIC (EDTA-free, pH 7.9). Lysates were centrifuged at 2000 ***g*** for 10 min at 4°C. Supernatant was then centrifuged at 100,000 ***g*** for 30 min at 4°C. Pellets were re-suspended in 1% NP-40 buffer. Protein concentration was determined by BCA (Pierce). Equal amounts of total protein were separated by SDS-PAGE using 7.5% or 15% Criterion Tris-Glycine gels (Bio-Rad), then transferred to Nitrocellulose membranes (Amersham). Membranes were blocked in 1× Tris-buffered saline (TBS) containing 5% non-fat dry milk and 0.01% Tween-20 for 1 h then incubated for 1 h with rabbit anti-Disp (1:15,000) or overnight at 4°C with rabbit anti-Dia (1:30,000) (Castrillon and Wasserman, 1994), rabbit anti-Hh (1:10,000, SCBT), rabbit anti-Kinesin (1:50,000, Cytoskeleton), mouse anti-actin (1:15,000, Millipore) or mouse anti-Tubulin (1:15,000, Cell Signaling), multiplexing when possible using appropriate secondary antibodies (Li-Cor IR and Jackson ImmunoResearch HRP, both at 1:15,000 dilution) for 30 min.

### Hh delivery efficiency

Delivery efficiency was calculated by counting the number of cells positive for background corrected Hh signal that were in contact with a Hh-containing cytoneme. The percentage of cells positive for Hh puncta was determined, and is shown as fold change relative to control dsRNA-treated cells.

### Drug treatment

Approximately 4×10^6^ S2 cells were plated and then transfected the following morning with 5 µg of *pAc-mCherry* using Lipofectamine 2000 (Thermo Fisher Scientific). Approximately 48 h post-transfection, cells were collected by centrifugation for 5 min at 250 ***g***, re-suspended in complete S2 media [10% Serum, 1× Pen/Strep Antibiotic, Schneider's Insect Medium (Sigma, S0146)] containing a 10 µM final concentration Nocodazole (Sigma), 10 µM final concentration cytochalasin D (Calbiochem) or DMSO control, and then immediately plated into chamber slides (∼1×10^6^ cells/well) and incubated for 1.5 h before MEM-fixation.

### Expression vectors and transgenes

CD8-mCherry was amplified from *pQUASp-mCD8mCherry* (Addgene) and inserted into *pAc5.1* using *Not*I and *Xba*I restriction sites to generate *pAc-mCD8-mCherry. pAC-DispHA* was generated by inserting *disp* PCR-amplified cDNA from *pFLC-I-disp* (DGRC) into a *pAc5.1-HA* using *Kpn*I restriction sites*.* Mutagenesis to make TM4 was performed using the QuikChange II XL mutagenesis kit (Agilent).

### Transgenic *Drosophila*

*Engrailed (en)-GAL4* and *UAS-CD8-RFP* were obtained from the Bloomington Stock Center. *UAS*-*dispHA*, *UAS-dispTM4HA* and *UAS-GFP* transgenic lines were generated using the PhiC31/attB system (Bischof et al., 2007). Transgenes were targeted to the ZH-51D landing site. Both male and female flies were assessed for phenotype. Male wings are shown in Fig. 6 and female wings in Fig. S3.

### Disp antibody

The coding region of the predicted fourth extracellular loop (amino acids 694-959) was introduced into *pET-28b* in frame with a carboxyl terminal 6× His tag. Protein was expressed in BL-21 cells and affinity purified on nickel resin by the St. Jude Protein Production Facility (Memphis, TN, USA). Antisera were produced in rabbits using Covance custom antibody service.
